# Contemporary Review of Masses in the Canal of Nuck

**DOI:** 10.7759/cureus.36722

**Published:** 2023-03-27

**Authors:** Hard Tilva, Surekha Tayade, Ankita Kanjiya

**Affiliations:** 1 Department of Obstetrics and Gynaecology, Jawaharlal Nehru Medical College, Datta Meghe Institute of Higher Education & Research, Wardha, IND; 2 Department of Obstetrics and Gynaecology, Sharda Hospital & Research Centre, Surat, IND

**Keywords:** the canal of nuck, cysts., tumors, endometriosis, hydrocele, hernia, inguinal hernia, female hydrocele, female, masses

## Abstract

Canal of Nuck masses is a rare occurrence that can cause swelling in the abdominal-inguinal region of females for various reasons. This condition arises due to an abnormal persistence of the processus vaginalis opening. Although Canal of Nuck pathology is not widely known among general surgeons or gynecologists due to its rarity, it has been associated with significant morbidity and requires further research. In this comprehensive review, we aim to summarize the embryology and anatomy of the Canal of Nuck, followed by a description of the various types of masses that can occur in this region. We discuss the clinical presentation and diagnostic workup of Canal of Nuck masses, including imaging modalities and differential diagnoses. Next, we review the surgical management of these masses, including open and laparoscopic approaches. Finally, we discuss the potential complications and long-term outcomes associated with Canal of Nuck pathology. This review aims to compile the presently accessible literature on anomalies occurring in the Canal of Nuck in females, with a particular focus on describing their pathological nature, diagnosis, and management. In summary, this review provides an up-to-date understanding of the pathology, diagnosis, and management of Canal of Nuck masses and aims to raise awareness of this under-recognized surgical challenge among healthcare providers.

## Introduction and background

The canal of Nuck was first identified by Dutch anatomist Anton Nuck in 1691 [1]. It is a developmental abnormality observed in females. The processus vaginalis remains enclosed within the inguinal canal, creating a potential space that connects the labia majora, the female inguinal canal, and the peritoneal cavity. While this phenomenon is commonly found in children, it is now increasingly detected in adults, likely due to the availability of advanced imaging modalities.

The inguinal canal is a narrow passage situated in the lower abdominal wall, measuring a mere 4 cm, and connecting the peritoneum to the perineum [2-7]. It runs from a superolateral to inferomedial direction and is lined by the aponeuroses of the external oblique, internal oblique, and transversus abdominis muscles [2-7]. The deep or internal inguinal ring is an oval opening in the transversalis fascia. In contrast, the superficial or external inguinal ring is a triangular opening in the aponeurosis of the external oblique muscle [2-7]. In females, these rings are less developed and may be absent altogether. The female inguinal canal also houses the ilioinguinal nerve and the round ligament of the uterus in addition to fat [2-7].

The inguinal canal's embryology involves the gubernaculum and the processus vaginalis. The gubernaculum, a muscular and fibrous tissue cord, forms between 8 and 12 weeks during fetal development [5]. In females, it develops into the round ligament of the uterus, which attaches to the middle portion of the uterus, passes through the inguinal canal, and connects to the labia majora [8]. Above this attachment, it becomes the ovarian suspensory ligament, which houses the ovarian vessels and prevents the ovary from descending into the inguinal canal [3,5,6]. The processus vaginalis is an invagination of the parietal peritoneum that descends anterior to the gubernaculum and is shorter in females than in males [5,6]. The superior part of the processus vaginalis obliterates at or soon before birth, and this obliteration continues caudally until the entire structure vanishes during the first year of life [2,4,6]. When there is a partial or total failure of obliteration of this processus vaginalis, the canal of Nuck forms as a potential space. In females, a patent processus vaginalis can result in direct communication with the inguinal canal, leading to hernias that may contain different organs and/or collections.

The most common clinical sign of canal of Nuck pathology is the presence of a mass or swelling in the groin or labia, which may be accompanied by pain. These masses encompass various differential diagnoses, including lymph node, cyst, inguinal hernia, infection/abscess, inguinal gonad, endometriosis, benign tumors, and neoplasia. Unfortunately, many clinicians, including surgeons, remain unaware of this anatomical variation and the potential pathology it may present. It is crucial for clinicians and surgeons to be knowledgeable about the masses in the canal of Nuck, as they can mimic other pathologies in the inguinal region. A thorough understanding of these masses' anatomy, clinical presentation, and imaging characteristics can help avoid misdiagnosis and inappropriate treatment. By making an accurate diagnosis, unnecessary biopsy or surgical intervention can be avoided, leading to better patient outcomes and reduced healthcare costs. Surgical intervention is considered the gold standard for managing symptomatic masses in the canal of Nuck. This involves carefully dissecting the mass and surrounding structures to minimize the risk of injury to adjacent nerves, vessels, and organs. In cases where malignancy is suspected, wide local excision with negative margins is recommended to achieve complete tumor removal. However, it is important to note that surgical management should be tailored to the individual patient and their specific clinical scenario. In some cases, conservative management with close observation may be appropriate, especially in asymptomatic or low-risk lesions.

## Review

Methods

We systematically searched PubMed and CENTRAL databases using specific keywords related to the canal of Nuck, masses, and anomalies in female patients. The search was limited to articles published within the last 10 years (2012-2022). We used a combination of the following keywords: 'canal of Nuck,' 'masses,' 'anomalies,' 'females,' 'women,' 'girls,' and 'pediatric.' Our inclusion criteria were studies that examined masses or anomalies in the canal of Nuck in female patients, regardless of age. We excluded studies that did not specifically focus on the canal of Nuck or masses/anomalies in female patients.

We initially retrieved 215,240 articles, which were then screened for relevance. After carefully reviewing and eliminating duplicate articles, we identified 1510 potentially relevant articles. We then used the inclusion and exclusion criteria to select the final articles for our review. We included studies that reported original research, case reports, or case series that specifically investigated the canal of Nuck, masses, or anomalies in female patients. We excluded studies that were not in English or did not report original data.

**Figure 1:**
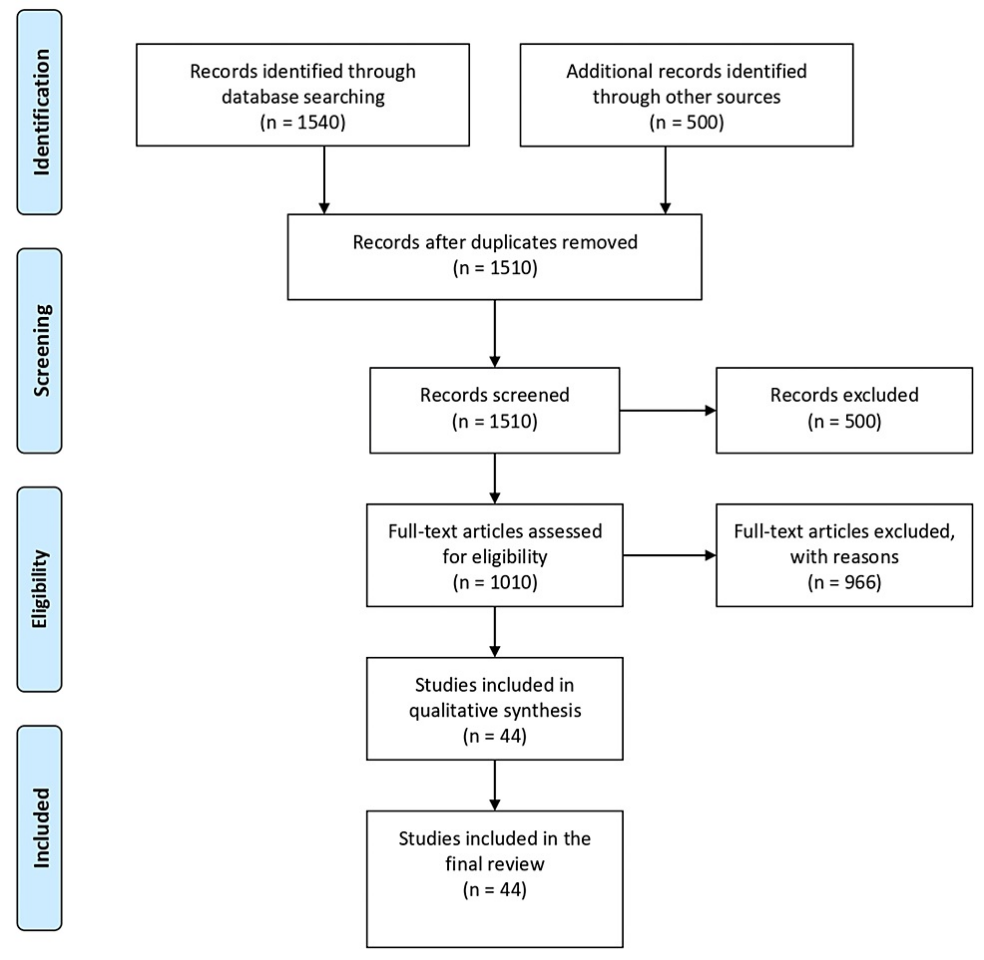
PRISMA flow diagram PRISMA = Preferred Reporting Items for Systematic Reviews and Meta-Analyses.

After applying these criteria, we identified 1010 relevant articles. We selected the final 44 studies from these articles for our qualitative review. The Preferred Reporting Items for Systematic Reviews and Meta-Analyses (PRISMA) flow diagram (Figure 1) illustrates the selection process of the relevant articles for our review. A detailed description of each of the studies included in our review is presented in Table 1.

**Table 1:** Articles included in the review

Author	Year	Place	Age of The Patient	Abnormality
Amu et al. [9]	2012	Enugu, Nigeria	18 Years	Epidermal inclusion dermoid cyst of canal of Nuck
Mandhan et al. [10]	2013	Muscat, Oman	5 Years	Infected hydrocele of canal of Nuck
Noguchi et al. [11]	2014	Saitama, Japan	45 Years	Ectopic pregnancy & Endometriosis of Cyst of canal of Nuck
Derinkuyu et al. [12]	2016	Ankara, Turkey	12 Weeks	Inguinal Hernia Containing Uterus, Bilateral Adnexa and Bowel
Sarkar et al. [13]	2016	West Bengal, India	3 Years	Hydrocele of Canal of Nuck
Choi and Baek [14]	2016	Yangsan, Republic of Korea	15 Days	Incarcerated ovarian herniation of the canal of Nuck
Sala et al. [15]	2017	Genova, Italy	17 Years	Giant Cyst of canal of Nuck
Uzun et al. [16]	2017	Edirne, Turkey	35 Years	Hemorrhagic cyst of the canal of Nuck, with the herniation of ligamentum rotundum into the inguinal canal
Ferreira et al. [17]	2017	Coimbra, Portugal	23 Years	Multiseptated Cyst Of canal Of Nuck
Shintaku et al. [18]	2018	Shiga, Japan	78 Years	Mullerian carcinosarcoma arising in hydrocele of the canal of Nuck
Topal et al. [19]	2018	Adana, Turkey	42 Years	Simple Cyst of canal of Nuck
Motooka et al. [20]	2018	Kumamoto, Japan	40 Years	Endometrioid carcinoma arising from endometriosis in the round ligament within the canal of Nuck
Shahid et al. [21]	2019	Doha, Qatar	36 Years	Hydrocele of canal Of Nuck
Zoorob et al. [22]	2019	Ottawa Hills, USA	52 Years	Vulvar pneumatocele Diagnosed during Robotic Hysterectomy
Baral et al. [23]	2020	Tansen, Nepal	25 Years	Bilateral Hydrocele of the canal of Nuck
Prodromidou et al. [24]	2020	Piraeus, Greece	40 Years	Cyst of canal of Nuck
Kumar et al. [25]	2020	Pune, India	4 Months	Herniation of the uterus, ovaries and fallopian tubes into the canal of Nuck
Chihara et al. [26]	2020	Kanagawa, Japan	38 Years	Hydrocele of the canal of Nuck in an Adult Female
Fukahori et al. [27]	2020	Kurume, Japan	7 Years	Cyst of the canal of Nuck with a ruptured abdominal cystic component
Kubo et al. [28]	2021	Kagoshima-ken, Japan	16 Days	Postnatal labial rupture by meconium peritonitis secondary to patent processus vaginalis
Hassan et al. [29]	2021	Washington, DC, USA	78 Years	Indirect hernia of canal of Nuck containing small bowel
Baig et al. [30]	2021	Saskatoon, Canada	70 Years	Cyst of canal of Nuck
Yeo and Tashi [31]	2021	Singapore, Singapore	35 Years	Round ligament varices presenting as a groin mass
Oshikiri et al. [32]	2022	Iwate, Japan	48 Years	Benign multicystic peritoneal mesothelioma in bilateral inguinal canal
Sadiqi et al. [33]	2022	Kabul, Afghanistan	6 Months	Incarcerated ovarian hernia in canal of Nuck
Vinoth et al. [34]	2022	Madhya Pradesh, India	36 Years	Hydrocele of canal of Nuck
Sofia et al. [35]	2022	Messina, Italy	38 Years	Pseudomyxoma peritonei involving the canal of Nuck

Discussion

While most pathological entities in the inguinal canal of females are typically found within the processus vaginalis, the canal of Nuck, some may arise from structures such as the ilioinguinal nerve or round ligament. However, for the purpose of this review, any abnormality detected in the inguinal canal of a female will be considered to be situated in the canal of Nuck. The most common types of masses in the canal of Nuck include hernias and hydroceles, infections or inflammatory conditions, vascular conditions, and neoplasms. Despite ongoing debates regarding the origin of these abnormalities, it is important to recognize the significance of their location within the canal of Nuck for accurate diagnosis and effective treatment.

Hernias and hydroceles

Canal of Nuck Hydrocele

The term "canal of Nuck hydrocele" refers to fluid accumulation in the canal of Nuck. This condition is indicated by swelling along the route of the inguinal canal or inside the labia majora. While it is usually not painful, it can cause discomfort if there is inflammation. There are three categories of the canal of Nuck hydrocele depending on the degree of fluid distension: Type 1 is a cyst of the canal of Nuck (also known as non-communicating or cystic hydrocele), Type 2 is a communicating hydrocele, and Type 3 is a bilocular hydrocele (also called an "hourglass hydrocele").

The most common type of canal of Nuck hydrocele is Type 1, which occurs due to incomplete occlusion of the canal. In this case, the caudal portion of the canal remains open while the cranial portion is occluded. This occlusion progresses from the deep to the superficial inguinal ring. Since there is no connection between the peritoneal cavity and the canal of Nuck, fluid accumulates in the canal, leading to the development of a cyst. A cystic dilation may be caused by a discrepancy between the absorption and secretion of fluid, an alteration in lymphatic drainage, trauma, or infection, or it may be idiopathic [36].

It should be noted that a canal of a Nuck cyst is different from an inguinal hernia sac in that it contains fluid instead of adipose tissue or intestine, and it is not connected to the peritoneal cavity. Type 2 of the cyst, a communicating hydrocele, is characterized by its continuity with the peritoneal cavity. As a result, the size and shape of the cyst may change during the Valsalva manoeuvres [37]. The third type, or "hourglass type," comprises two cysts, one linked to the superior peritoneal cavity. This "bilocular" appearance is caused by a narrow, deep inguinal ring that allows only partial closure [38].

Sarkar et al. reported a case of encysted hydrocele in a three-year-old female [13]. Sala et al. also documented a case of a giant cyst of the Nuck canal in a 17-year-old female who presented with a 10x4 cm mass arising from the labia majora [15]. Other reported cases of these hydroceles and cysts are included in (Table 1) [17,19,21,23,24,26,30,34].

Canal of Nuck Hernia

A patent processus vaginalis increases the likelihood of acquiring an indirect inguinal hernia. In this situation, when any intra-abdominal organ herniates through the deep inguinal ring, lateral to the inferior epigastric artery, following the round ligament and ultimately reaching the labia majora, the hernia is categorized as "indirect" [39]. Hernias of the canal of Nuck is relatively more common in preterm infants, as the canal of Nuck typically closes within the first year of life [2-4,6]. It is common for the ovary to herniate through the inguinal canal in tubal and ovarian hernias cases, specifically in the pediatric population, when the gubernaculum is not attached to the uterus. Derinkuyu et al. described one such instance involving a female infant of 12 weeks, which had a hernia consisting of the uterus, bilateral adnexa, and colon [12]. Kumar et al. presented a case in which a four-month-old female infant's uterus, ovaries, and fallopian tubes herniated into the canal of Nuck [25]. Hassan et al. reported a case involving a 78-year-old woman with a massive canal of Nuck Hernia containing small bowel loops [29].

These types of hernias have a high morbidity rate due to the high risk of incarceration and torsion, which can lead to necrosis. Choi and Baek reported a case of a 15-day-old girl with an ovarian hernia that was incarcerated in the canal of Nuck [14]. Sadiqi et al. also reported a case of a six-month-old girl with an incarcerated ovarian hernia in the canal of Nuck, which was also strangulated [33].

Ultrasonography (USG) is generally the first imaging modality to diagnose hernias in children, while computed tomography (CT) scans are often used in adults with an acute abdomen. However, surgeons must exercise caution when dealing with adult hernias, particularly if they are painful or complex. These can be associated with a higher risk of complications such as intestinal blockages or ischemia. In severe cases, these blockages can lead to symptoms such as nausea, vomiting, abdominal pain, and constipation. Surgeons must remain vigilant for signs of complications and consider prompt intervention to minimize the risk of serious harm [14,33].

Inguinal Gonads

Hernias in the inguinal canal of females can contain either the ovaries alone or the ovary in conjunction with other reproductive organs. Inguinal hernias containing the ovary are more common in newborns than adolescents or adults, who encounter them relatively less frequently. These types of hernias may be more prevalent in preterm newborns, as the processus vaginalis fuses within the first year of life, leaving them with a patent processus vaginalis. Inguinal hernias in females can also be indirect or direct, with indirect hernias being more common and often associated with a patent processus vaginalis. Regardless of their type, hernias in the inguinal canal can be a significant source of morbidity and require prompt diagnosis and treatment.

Infectious or inflammatory conditions

Inflammatory or infectious diseases of the canal of Nuck can arise from the hematogenous spread of an existing phlogistic process into a pre-existing cyst in the canal of Nuck. Alternatively, they may be linked to an abdominal infection that spreads into the canal due to its communication with the peritoneal cavity. This clinical manifestation is typically marked by pain or discomfort, feverish illness, an inguinal lump, and reactive immunological tests.

Cysts of Canal of Nuck

Although rare, cysts in the canal of Nuck are the most commonly reported pathology [4]. A cyst is formed when the proximal end of the processus vaginalis fuses, leaving a portion distally open. This is a genuine cyst, not a pseudocyst, as it is a specific section of the processus vaginalis lined by epithelial cells.

Due to their elongated and narrow form, cysts in the canal of Nuck are often referred to as having a sausage-like appearance. In one case, Ferreira et al. reported a 23-year-old girl with a sausage-shaped lump in her right labia majora, which was a cyst of the canal of Nuck [17].

On the other hand, if fluid accumulates within the cyst, it can expand and become more spherical. Similar to cysts in other parts of the body, a Nuck canal cyst can be simple or complex with internal septations and debris. In a case report by Lai et al., a complex cyst was reported to have formed in the canal of Nuck [40].

Surgery is the only successful treatment for a cyst in the canal of Nuck. Many patients with symptomatic cysts undergo surgery to treat their symptoms or because a hernia was assumed to be the underlying condition. Simple, asymptomatic cysts can be treated with percutaneous aspiration, but recurrence has been documented [36].

Infection/Abscess

Given its anatomical relationship with the peritoneal cavity, infections such as abscesses are typical of intra-abdominal origin. However, pathologies within the canal of Nuck, such as cysts, may also become superinfected via hematogenous dissemination, leading to an abscess within the canal. Clinical manifestations of such abscesses may include fever, discomfort, the presence of an inguinal mass, and/or elevated levels of inflammatory markers, including leukocytosis. Similar to the management of abscesses in other areas of the body, treatment may involve the use of antibiotics and drainage.

Endometriosis

Endometriosis is a condition that frequently occurs in the ovary, pelvic portion of the parietal peritoneum, pouch of Douglas (POD), and uterosacral ligament [41]. There are currently three hypotheses surrounding its development [42]. The metastatic hypothesis proposes that endometrial tissue is deposited on serosal surfaces during retrograde menstruation. The metaplastic theory suggests that peritoneal epithelial cells can transform into endometrial cells. The induction hypothesis postulates that the release of chemicals by the shed endometrium signals the surrounding mesenchyme to convert into endometrial tissue. Although individuals may not exhibit any symptoms, pelvic discomfort is a common cyclical feature of the classic clinical presentation. Endometrial implantation in the canal of Nuck, especially at the attachment of the round ligament, is often accompanied by painful lumps in the inguinal region [41].

The most common type of endometrial implant seen in the canal of Nuck is a solid, fibrotic endometrioma, which is more frequently identified on the right side [41]. Surgical resection is the most commonly used treatment for endometriosis, particularly for symptomatic implants [42]. Typically, these implants in the canal of Nuck are discovered when patients experience sudden or recurring pain, which may require surgical correction. However, hormonal therapy can treat tiny implants medically [42]. In a report by Noguchi et al. [11], a 45-year-old Japanese woman was diagnosed with endometriosis and an ectopic pregnancy in a cyst of her canal of Nuck.

Vascular conditions

Commonly, this condition involves hematomas, varicoceles, and, in rare cases, post-traumatic arteriovenous fistulas, aneurysms, and varices of the great saphenous vein.

Hematoma

An inguinal hematoma typically presents as soreness and swelling in the affected area. In addition to post-traumatic or postsurgical causes, anticoagulation therapy, catheter administration, and malignancy can also cause hematomas [7]. Hematomas are more commonly found in the inguinal area near the femoral vessels rather than within the inguinal canal itself [4]. While they often resolve independently without treatment, drainage may be necessary if symptoms are present [7].

Thorgersen et al. reported a hematoma in the inguinal canal of a 42-year-old male patient previously treated with warfarin [43]. To our knowledge, no similar cases have been reported in females, which highlights the need for further research and investigation into the sex-specific differences in the presentation and management of inguinal canal hematomas.

Varicocele

Generally, when the term "female varicocele" is used, it refers to pelvic congestion syndrome. Nonetheless, a varicocele can occur in the canal of Nuck, which is located near the round ligament [44]. These varicoceles have enlarged veins that expand or become engorged during the Valsalva maneuver. Such varicoceles were historically observed in pregnant women, particularly those in the advanced stages of pregnancy. A physical examination may misdiagnose a varicocele as an inguinal hernia. However, surgery can be avoided if diagnosed correctly since the symptoms tend to disappear spontaneously after delivery, and no further treatment is required [44]. Yeo and Tashi documented round ligament varices associated with pregnancy in a 35-year-old woman [31].

Neoplasms

Neoplasms can originate from various types of cells, such as fat, muscle, blood vessels, connective tissue, neural tissue or lymphoid tissue, all of which are structural components of the inguinal canal. Similar to other body parts, these neoplasms can be benign or malignant. Among the benign lesions, a lipoma, a tumor composed entirely of fat, is the most prevalent [4]. However, it is frequently misdiagnosed with a hernia that contains fat and a lipoma. In the case of a genuine canal of Nuck lipoma, there is no direct connection to the intraperitoneal or retroperitoneal fat.

Various benign masses can appear in the canal, including neurofibromas, desmoid tumors, and leiomyomas. Desmoid tumors are fibrous tumors that are both aggressive and benign. Although many of the structures in the canal of Nuck can give rise to primary malignant neoplasms, it is extremely rare for benign conditions to transform into malignant forms. For instance, an endometrial implant can lead to endometroid cancer [42]. The canal of Nuck can potentially contain metastatic lesions as well [4,7]. However, since metastases typically spread through a lymphatic or hematogenous pathway, they are not directly related to nearby tissues. The most common approach for treating benign tumors is surgically removing them [7]. Surgical removal is also an option for primary malignant tumors of the canal of Nuck without evidence of metastasis. Chemotherapy is usually the most utilized treatment modality for tumors that have spread to the canal of Nuck. One such unique case is pseudomyxoma peritonei, which is treated using cytoreduction and debulking surgery followed by intraperitoneal chemotherapy [7].

Sofia et al. reported a single instance of pseudomyxoma peritonei that affected the canal of Nuck. The case involved a 38-year-old woman who suffered from infertility and chronic pelvic pain [35]. On the other hand, Motooka et al. described a case of endometrioid carcinoma in a 40-year-old woman who had endometriosis in the round ligament inside the canal of Nuck [20]. Another case of Mullerian carcinosarcoma with neuroendocrine differentiation was reported by Shintaku et al. in a 78-year-old female with a hydrocele of the canal of Nuck [18].

Results

This review article aims to identify the various pathological entities that arise in the inguinal canal of females, focusing on the canal of Nuck. The most common abnormalities detected in this canal include hernias and hydroceles, infections or inflammatory conditions, vascular conditions, and neoplasms.

The canal of Nuck hydrocele is indicated by swelling along the route of the inguinal canal or inside the labia majora. There are three categories of the canal of Nuck hydrocele depending on the degree of fluid distension, including Type 1 cyst of the canal of Nuck, Type 2 communicating hydrocele, and Type 3 bilocular hydrocele. Type 1 is the most common type of canal of Nuck hydrocele and is caused due to incomplete occlusion of the canal, leading to fluid accumulation in the canal [7].

Hernias of the canal of Nuck is relatively more common in preterm infants, as the canal of Nuck typically closes within the first year of life. The ovary may herniate through the inguinal canal in tubal and ovarian hernias, specifically in the pediatric population, when the gubernaculum is not attached to the uterus. These types of hernias have a high morbidity rate due to the high risk of incarceration and torsion, which can lead to necrosis [13].

Ultrasonography (USG) is generally the first imaging modality to diagnose hernias in children, while computed tomography (CT) scans are often used in adults with an acute abdomen. Surgeons must exercise caution when dealing with adult hernias, particularly if they are painful or complex. These can be associated with a higher risk of complications such as intestinal blockages or ischemia [31]. 

Overall, recognizing the significance of the location of these abnormalities within the canal of Nuck is important for accurate diagnosis and effective treatment. The review article provides a comprehensive overview of the various pathological entities that arise in the inguinal canal of females and highlights the need for prompt intervention to minimize the risk of serious harm.

## Conclusions

In conclusion, canal of Nuck masses is a rare but clinically significant condition that requires timely recognition and appropriate management. Diagnosis can be challenging, but a thorough understanding of the anatomy and diagnostic workup is crucial to accurately identify and differentiate these masses from other pelvic lumps or swellings. Once diagnosed, canal of Nuck masses management often involves surgical intervention, with both open and laparoscopic approaches available. With advances in imaging technology and surgical techniques, the prospects for successfully managing these masses have significantly improved in recent years. It is important to raise awareness of this under-recognized surgical challenge among healthcare providers to ensure patients receive timely and appropriate management.

## References

[1] Antonius Nuck, Maurits van Reverhorst. Nuck A (2023). Adenographia curiosa et uteri foeminei anatome nova. Adenographia curiosa et uteri foeminei anatome nova. apud Samuelem Luchtmans; 1722.

[2] Bhosale PR, Patnana M, Viswanathan C, Szklaruk J (2008). The inguinal canal: anatomy and imaging features of common and uncommon masses. Radiographics.

[3] Sameshima YT, Yamanari MG, Silva MA, Neto MJ, Funari MB (2017). The challenging sonographic inguinal canal evaluation in neonates and children: an update of differential diagnoses. Pediatr Radiol.

[4] Laing FC, Townsend BA, Rodriguez JR (2007). Ovary-containing hernia in a premature infant: sonographic diagnosis. J Ultrasound Med.

[5] Shadbolt CL, Heinze SB, Dietrich RB (2001). Imaging of groin masses: inguinal anatomy and pathologic conditions revisited. Radiographics.

[6] Patel B, Zivin S, Panchal N, Wilbur A, Bresler M (2014). Sonography of female genital hernias presenting as labia majora masses. J Ultrasound Med.

[7] Revzin MV, Ersahin D, Israel GM, Kirsch JD, Mathur M, Bokhari J, Scoutt LM (2016). US of the inguinal canal: comprehensive review of pathologic processes with CT and MR imaging correlation. Radiographics.

[8] Clarnette TD, Hutson JM (1999). The development and closure of the processus vaginalis. Hernia 3.

[9] Amu OC, Udeh EI, Ugochukwu AI, Madu C, Nzegwu MA (2012). A case of vulval swelling secondary to female circumcision posing a diagnostic dilemma. Int J Surg Case Rep.

[10] Mandhan P, Raouf Z, Bhatti K (2013). Infected hydrocele of the canal of Nuck. Case Rep Urol.

[11] Noguchi D, Matsumoto N, Kamata S, Kaneko K (2014). Ectopic pregnancy developing in a cyst of the canal of Nuck. Obstet Gynecol.

[12] Derinkuyu BE, Affrancheh MR, Sönmez D, Koloğlu MB, Fitoz S (2016). Canal of Nuck hernia in a female infant containing uterus, bilateral adnexa and bowel. Balkan Med J.

[13] Sarkar S, Panja S, Kumar S (2016). Hydrocele of the canal of Nuck (female hydrocele): a rare differential for inguino-labial swelling. J Clin Diagn Res.

[14] Choi KH, Baek HJ (2016). Incarcerated ovarian herniation of the canal of Nuck in a female infant: ultrasonographic findings and review of literature. Ann Med Surg (Lond).

[15] Sala P, Palmeri A, Costantini S (2018). Giant cyst of the Nuck canal: a worrisome trouble for a girl. Am J Obstet Gynecol.

[16] Uzun I, İnan C, Varol F, Erzincan S, Sütcü H, Sayin C (2017). Hemorrhagic cyst of the canal of Nuck after vaginal delivery presenting as a painful inguinal mass in the early postpartum period. Eur J Obstet Gynecol Reprod Biol.

[17] Ferreira AF, Marques JP, Falcão F (2017). Hydrocele of the canal of Nuck presenting as a sausage-shaped mass. BMJ Case Rep.

[18] Shintaku M, Kamada Y, Sumitomo M (2018). Müllerian carcinosarcoma with neuroendocrine differentiation arising in hydrocele of the canal of Nuck. Pathol Int.

[19] Topal U, Sarıtaş AG, Ülkü A, Akçam AT, Doran F (2018). Cyst of the canal of Nuck mimicking inguinal hernia. Int J Surg Case Rep.

[20] Motooka Y, Motohara T, Honda R, Tashiro H, Mikami Y, Katabuchi H (2018). Radical resection of an endometrioid carcinoma arising from endometriosis in the round ligament within the right canal of Nuck: a case report and literature review. Gynecol Oncol Rep.

[21] Shahid F, El Ansari W, Ben-Gashir M, Abdelaal A (2020). Laparoscopic hydrocelectomy of the canal of Nuck in adult female: case report and literature review. Int J Surg Case Rep.

[22] Zoorob D, Spalsbury M, Slutz T, Oliver M, Tsolakian I, Judis J (2019). Unilateral vulvar pneumatocele (pneumolabium) diagnosed during robotic hysterectomy. Case Rep Obstet Gynecol.

[23] Baral S, Bajracharya P, Thapa N, Chhetri RK (2020). Bilateral hydrocele of the canal of Nuck: a rare presentation in an adult female. Int Med Case Rep J.

[24] Prodromidou A, Paspala A, Schizas D, Spartalis E, Nastos C, Machairas N (2020). Cyst of the canal of Nuck in adult females: a case report and systematic review. Biomed Rep.

[25] Kumar D, Maheshwari S, Rajesh U, Grewal D, Maria V (2020). Herniation of the uterus, ovaries and fallopian tubes into the canal of Nuck in a 4-month-old child: A rare entity. SA J Radiol.

[26] Chihara N, Taniai N, Suzuki H, Nakata R, Shioda M, Yoshida H (2020). Use of a novel open posterior wall technique for laparoscopic excision of hydrocele of the canal of Nuck in an adult female: case report. J Nippon Med Sch.

[27] Fukahori S, Sakamoto S, Hashizume N (2021). Laparoscopic identification of combined pediatric femoral hernia and ruptured abdominal cyst of the canal of Nuck: a report of an extremely rare case. Asian J Endosc Surg.

[28] Kubo Y, Sugita K, Ibara S (2021). The female neonate who showed postnatal labial rupture by meconium peritonitis. Pediatr Int.

[29] Hassan M, Stutsrim AE, J Clark C (2021). Large canal of Nuck hernia: the female equivalent of the inguinoscrotal hernia. Am Surg.

[30] Baig Z, Hunka N, Gaboury J (2021). Surgical treatment of a canal of Nuck cyst presenting as a femoral hernia: an unusual case report. Int J Surg Case Rep.

[31] Yeo JH, Tashi S (2021). A Rare but noteworthy diagnosis for "lumps in the groin" during pregnancy: round ligament varices. Am J Case Rep.

[32] Oshikiri H, Ozawa Y, Suzuki O, Usuda M, Miyata G (2022). Benign multicystic peritoneal mesothelioma occurring in bilateral inguinal canals metachronously: a case report. Surg Case Rep.

[33] Sadiqi J, Ezmarai M, Niazi J (2022). Canal of Nuck incarcerated ovarian hernia with strangulation, a case report. Radiol Case Rep.

[34] Vinoth T, Lalchandani A, Bharadwaj S, Pandya B (2022). Revisiting the clinico-radiological features of an unusual inguino-labial swelling in an adult female. Int J Surg Case Rep.

[35] Sofia C, Marino MA, Milone E, Ieni A, Blandino A, Ascenti G, Macrì A (2022). Pseudomyxoma peritonei involving the canal of Nuck: The added value of magnetic resonance imaging for detection and presurgical planning. Radiol Case Rep.

[36] Stickel WH, Manner M (2004). Female hydrocele (cyst of the canal of Nuck): sonographic appearance of a rare and little-known disorder. J Ultrasound Med.

[37] Zawaideh JP, Trambaiolo Antonelli C, Massarotti C, Remorgida V, Derchi LE (2018). Cyst of Nuck: a disregarded pathology. J Minim Invasive Gynecol.

[38] Counseller VS, Black BM (1941). Hydrocele of the canal of Nuck: report of seventeen cases. Ann Surg.

[39] Husaric E, Hotic N, Halilbasic A, Husaric S, Rahmanovic E, Suljendic S (2014). Cyst of the canal of Nuck in a two year old girl. Med Arch.

[40] Lai I, Page A, Hamidinia F, Rahmani R (2017). Cysts of the canal of Nuck: a rare sonographic diagnosis. J Clin Ultrasound.

[41] Gaeta M, Minutoli F, Mileto A, Racchiusa S, Donato R, Bottari A, Blandino A (2010). Nuck canal endometriosis: MR imaging findings and clinical features. Abdom Imaging.

[42] Woodward PJ, Sohaey R, Mezzetti TP Jr (2001). Endometriosis: radiologic-pathologic correlation. Radiographics.

[43] Thorgersen EB, Øfsti AM, Nyheim T, Abedini S (2002). Acute groin pain and femoral nerve deficit in a warfarin treated patient (Article in Norwegian). Tidsskr Nor Laegeforen.

[44] Murphy IG, Heffernan EJ, Gibney RG (2007). Groin mass in pregnancy. Br J Radiol.

